# HIF-2α Suppresses p53 to Enhance the Stemness and Regenerative Potential of Human Embryonic Stem Cells

**DOI:** 10.1002/stem.1142

**Published:** 2012-08

**Authors:** Bikul Das, Reza Bayat-Mokhtari, Micky Tsui, Shamim Lotfi, Rika Tsuchida, Dean W Felsher, Herman Yeger

**Affiliations:** a Division of Oncology, Departments of Medicine and Pathology, Stanford University School of MedicineStanford, California, USA; b Division of Hematology and Oncology, Department of Paediatrics, The Hospital for Sick Children, University of TorontoToronto, Ontario, Canada; c Developmental Biology and Stem Cell Program, The Hospital for Sick Children, University of Toronto, TorontoOntario, Canada; d Department of Paediatric Laboratory Medicine, The Hospital for Sick Children, University of TorontoToronto, Ontario, Canada; e Department of Molecular Oncology, Tokyo Medical and Dental UniversityTokyo, Japan

**Keywords:** Embryonic stem cells, altruism, Hypoxia, Neoplastic stem cell biology, Reprogramming, Self-renewal, Oxidative stress

## Abstract

Human embryonic stem cells (hESCs) have been reported to exert cytoprotective activity in the area of tissue injury. However, hypoxia/oxidative stress prevailing in the area of injury could activate p53, leading to death and differentiation of hESCs. Here we report that when exposed to hypoxia/oxidative stress, a small fraction of hESCs, namely the SSEA3+/ABCG2+ fraction undergoes a transient state of reprogramming to a low p53 and high hypoxia inducible factor (HIF)-2α state of transcriptional activity. This state can be sustained for a period of 2 weeks and is associated with enhanced transcriptional activity of Oct-4 and Nanog, concomitant with high teratomagenic potential. Conditioned medium obtained from the post-hypoxia SSEA3+/ABCG2+ hESCs showed cytoprotection both in vitro and in vivo. We termed this phenotype as the “enhanced stemness” state. We then demonstrated that the underlying molecular mechanism of this transient phenotype of enhanced stemness involved high Bcl-2, fibroblast growth factor (FGF)-2, and MDM2 expression and an altered state of the p53/MDM2 oscillation system. Specific silencing of HIF-2α and p53 resisted the reprogramming of SSEA3+/ABCG2+ to the enhanced stemness phenotype. Thus, our studies have uncovered a unique transient reprogramming activity in hESCs, the enhanced stemness reprogramming where a highly cytoprotective and undifferentiated state is achieved by transiently suppressing p53 activity. We suggest that this transient reprogramming is a form of stem cell altruism that benefits the surrounding tissues during the process of tissue regeneration.

## INTRODUCTION

Numerous studies have revealed that although the majority of transplanted stem cells die, and do not engraft, stem cells secrete growth factors and antioxidants that likely contribute to the repair and regeneration of the inflamed and damaged tissues [1]. This view is consistent with the findings that undifferentiated stem cells are capable of secreting high levels of antioxidants and growth factors [2]. However, the site of injury/inflammation is particularly characterized by hypoxia and production of reactive oxygen species (ROS). The ROS could induce DNA damage, and subsequently trigger p53 activation in stem cells, leading to differentiation and apoptosis [3]. Hence, it is suggestive that stem cells may have evolved specific mechanisms to maintain an undifferentiated state in the area of injury to serve their cytoprotective activity. Therefore, we posited that stem cells might have acquired molecular mechanisms to suppress p53 and remain functionally undifferentiated during hypoxia and oxidative stress in order to be able to conduct their cytoprotective activity.

One of the potential ways that stem cells could suppress p53 is by activating HIF-2α. Hypoxia upregulates the key transcription factors HIF-1α and HIF-2α with numerous downstream targets that play critical roles in survival, angiogenesis, metabolism, and other important functions [4]. HIF-1α stabilizes p53, and therefore enhances p53 activation [5], whereas HIF-2α is shown to suppress p53 activity by reducing ROS generation [6]. HIF-2α also upregulates several genes involved in antioxidant mechanisms including *SOD*, *catalase*, *GSPx* [7], and *ABCG2* [8]. ABCG2 is an efflux pump [9–12], which is found to secrete glutathione (GSH) [13], a primary antioxidant and component of the GSH/glutathione-s-transferase (GST) detoxification system [14]. HIF-2α also enhances the secretion of growth factors having cytoprotective and regenerative abilities, for example, vascular endothelial growth factor (VEGF) and fibroblast growth factor (FGF)-2 [15]. Thus, one potential mechanism of p53 suppression would be to enhance HIF-2α expression, which could not only contribute to protect the stem cells from p53-induced differentiation or death but also enhance their cytoprotective activity. Therefore, we sought evidence that stem cells may suppress p53 activity during hypoxia and oxidative stress by upregulating HIF-2α in order to exert their cytoprotective and regenerative activities. We also wanted to determine whether the same mechanism might also be involved in the potential transformation of stem cells to cancer stem cells as has been recently documented [16, 17]

To investigate the above possibilities, we used human embryonic stem cells (hESCs) as a model cell type. There are several advantages in using ESCs to test our hypothesis. First, p53 is highly active in hESCs and second, it directly inhibits Nanog, one of the core regulators involved in maintaining the stemness state [18–20]. Third, HIF-2α is active in ESCs [15]. We exposed hESCs to an extreme environment of hypoxia and oxidative stress and applied a novel approach to isolate a surviving, undifferentiated hESC fraction having low p53 and high HIF-2α activity. We were successful in isolating a small fraction of hESCs, the SSEA3+/ABCG2+ fraction, which exhibited high HIF-2α and low p53 activity as well as enhanced cytoprotective activity. While investigating the undifferentiated state, we serendipitously discovered that the SSEA3+/ABCG2+ cells undergo a transient state of reprogramming to an even higher state of stemness, here termed the “enhanced stemness” state, having very high Nanog expression, high antioxidant secretion, and high cytoprotective activity following exposure to hypoxia and oxidative stress.

## MATERIALS AND METHODS

### Reagents and Chemicals

All the chemicals were obtained from Sigma-Aldrich (St. Louis, MO, http://www.sigmaaldrich.com) unless otherwise noted. The HIF-1α/2α inhibitor FM19G11 [21] was obtained from Millipore (Billerica, MA, http://www.millipore.com [#400089-10MG]), Pifithrin-α from ChemBridge Corporation (San Diego, CA, http://www.chembridge.com), caspase-3 substrate, *N*-acetyl-DEVD-7-amino-4-methylcoumarin peptide from Enzo Life Sciences (Farmingdale, NY, http://www.enzolifesciences.com) [22] and recombinant FGF-2 and Matrigel from R&D Systems (Minneapolis, MN, http://www.rndsystems.com). All the culture media reagents were obtained from Invitrogen Technologies (CA, Invitrogen Life Technologies, Grand Island, NY, http://www.invitrogen.com) until otherwise noted.

### hESC Culture and Induction of Hypoxia

Due permissions were obtained to culture hESCs both at Department of Medicine, Stanford University (Stem Cell Research Oversight protocol #285, director: BD) and at Department of Laboratory Medicine and Pathobiology, Hospital for Sick Children. BGO1 and H9 hESCs (WiCell Inc., Madison, WI, http://www.wicell.org) were expanded on Mitomycin-C (10 μg/ml; Sigma)-inactivated mouse embryonic fibroblast (MEF) feeders, cultured in knockout Dulbecco's modified Eagle's medium/F12 with 20% knockout serum replacement (KOSR, #12660012, and #10828-028; Invitrogen Life Technologies, Grand Island, NY), and 4 ng/ml FGF-2 (#PHG0024, Gibco, Life Technologies, Grand Island, NY, http://www.invitrogen.com) as described elsewhere [23, 24]. The expanded hESCs were then grown on feeder-free Matrigel (##354230, BD Biosciences, San Jose, CA, http://www.invitrogen.com)-coated dishes with MEF-derived conditioned media (CM), supplemented with 8 ng/ml FGF-2 (complete hESC media), and under standard conditions (37°C and 5% CO_2_ and saturated humidity) as previously described [25] and used to perform various experiments described in the text. To make single-cell suspension for flow cytometry and to perform plating efficiency experiments, the hESCs were dispersed with TrypLe select (#12563-011, Invitrogen) for 3 minutes and then washed twice with the complete hESC media supplemented with 10 μM Rho-associated protein kinase inhibitor Y-27632 (Calbiochem, Merck, Rockland, MA, http://www.emd.us). The hESCs were then suspended in the complete media plus Rho-associated protein kinase (ROCK) inhibitor to maintain viability during flow cytometry sorting [26]. For the hypoxia experiments, day-2 hESCs culture having 30%–40% confluence was used and hypoxia was achieved as described previously [22, 23]. Briefly, hypoxic condition (<0.1% O_2_) was established in a sealed chamber using the BBL GasPak plus anaerobic system envelopes with a palladium catalyst (Becton Dickinson, Cockeysville, MD, http://www.bd.com/ds). Immediately after hypoxia, the cells were incubated in tissue culture incubator at 37°C and 5% CO_2_. Medium was changed only 24 hours after hypoxia so that the cultured cells were exposed to ROS generated immediately during the reoxygenation process [27].

### Flow Cytometry

Flow cytometry was performed using FACScan and FACSAria II (BD Bioscience) as described [23, 28]. Surface staining and flow cytometry sorting was done as described elsewhere [23]. Briefly, the single-cell suspensions of hESCs were incubated with primary antibodies (Supporting Information Table 1) in staining media (phosphate buffered saline (PBS) with 2% KOSR, Invitrogen), on ice, for 15 minutes, washed thrice in staining medium, and then incubated with Alexafluor secondary antibody for 30 minutes. After washing, the stained cells were suspended in complete culture media and kept at 4°C.

### Immunostaining

Cells were fixed in 4% paraformaldehyde (Electron Microscopy Sciences, Hatfield, PA, http://www.emsdiasum.com) for 10 minutes on ice, permeabilized, washed thrice in PBS, and incubated overnight in staining buffer (4% bovine serum albumin (BSA) + 0.1% saponin) with primary antibodies against HIF-2α and p53 (Supporting Information Table 1). They were then washed and incubated with secondary Alexa 488 or 594 antibodies for 1 hour at RT. For immunostaining of teratoma tissues, frozen sections were fixed with 4% paraformaldehyde, permeabilized with 0.1% saponin, and subjected to immunostaining as described elsewhere [23].

### Cell Viability and Apoptosis Measurement

The hESCs were grown onto Matrigel-coated dishes in complete hESCs media. At day 2 after plating, cells were exposed to hypoxia for 24 hours and then reoxygenation for next 4 days. On day-4 post-hypoxia, the viability was determined by Trypan blue assay [22]. A portion of the cells was subjected to flow cytometry sorting, and then the sorted population was subjected to caspase-3 activity assay as previously described [22].

### ELISA Assay

The cell lysates were subjected to ELISA assay as previously described [22]. Information about various ELISA kits used in the study is given in Supporting Information Table 1.

### InCell Western Assay

The assay was performed as per manufacturer instructions (Licor Bioscience, NE, Lincoln, NE, http://www.licor.com). The hESCs (5 × 10^3^ per well) were plated in sterile 96-well plates (BG Falcon, #353075) and kept at RT for 30 minutes before transfer to the incubator to reduce excessive settling at the well edges. The plates were incubated overnight at 37°C and then fixed with 3.7% formaldehyde at RT for 10 minutes. After fixation, the plates were washed with PBS, then primary antibodies were added at 1:300 concentration in 2% BSA + 0.1% saponin, and incubated overnight at 4°C. After primary antibody incubations, plates were washed thrice (5 minutes each) with 100 μl per well PBS-T (PBS with Tween 20) at RT. Then, secondary antibodies conjugated to IRDye 800CW (goat-anti-rabbit-IgG (Licor Biosciences, NE) or goat-anti-mouse-IgG (Licor Biosciences, NE) were used at 1:1,000 dilution. The nuclear staining dye DRAQ5 (Biostatus Ltd., Leicestershire, U.K., http://www.biostatus.com) combined with Sapphire700 (Li-COR) at 1:10,000 and 1:1000, respectively, were also added. Plates were then incubated with 50 μl per well secondary antibody solutions for 90 minutes at R/T in the dark. Background control wells were prepared by omitting primary antibodies (i.e., secondary only). After secondary antibody incubations, plates were washed thrice times (10 minutes each) with PBS-T at R/T in the dark, and then plates were dried in air before scanning. Plates were scanned and analyzed using an Odyssey IR scanner using Odyssey imaging software 3.0. Scan settings used were medium or high image quality, 169 μm resolution, intensity 5.0 for the both the 700-channel and 800-channel with an offset of 4.0 mm. For signal quantification, antibody signals were analyzed as the average 800-channel integrated intensities from triplicate wells normalized to the 700-channel signal integrated intensity to correct for well-to-well variations in cell number. Results are expressed as fold change (means ± standard errors of the mean) compared to noninfected controls.

### Measurement of Transcription Factor Activity

The transcriptional activity of HIFs and p53 were measured by a transcription factor (TF) filter play assay system developed by Signosis, Sunnyvale, CA, http://www.signosisinc.com (Supporting Information Table 1). The assay was performed as per manufacturer instructions. Briefly, the nuclear extracts of hESCs were mixed with a specific biotin-labeled TF DNA binding sequence to obtain TF-DNA complexes. Then, the TF-DNA complexes were retained in a filter plate and hybridization was achieved. The captured DNA probe was quantified by streptavidin-horseradish peroxidase (HRP) method on a microplate luminometer. The Oct-4 and Nanog transcriptional activities were measured by Cignal Lenti Nanog reporter (Luciferase) kit (SABioscience, Valencia, CA, http://www.sabio.sciences.com) as per manufacturer instructions.

### Measurement of Oxidative Stress

The level of ROS was measured by DCFH-DA assay as described [23]. The oxidative DNA damage was measured by a 8-hydroxy-2-deoxy Guanosine (8-OH-dG) Enzyme Immuno Assay kit (#589320; Cayman Chemical, MI, Ann Arbor, MI, http://www.caymanchem.com) as per manufacturer's instructions.

### GSH Measurement

The GSH was measured using the glutathione assay kit (#GT10, Oxford Biomedical Research, MI, http://www.oxfordbiomed.com) as described previously [14].

### siRNA Gene Silencing

We performed small interfering RNA (siRNA) gene silencing by Accell siRNA (Thermoscientific Dharmacon, Lafayette, CO, USA, http://www.dharmacon.com). The following Accell siRNAs were used: the human HIF-2α (A-004814-17-0005), HIF-1α (A-004018-24-0005), and p53 (A-003329-22-0005). The hESCs (5 × 10^3^ per well in 96-well plate) were cultured in the completed hESC media, and 1 μM Accell siRNA was delivered to each well as per manufacturer instructions. After incubation for 72 hours at 37°C, the media were replaced with fresh media, and the cells were exposed to hypoxia. The gene knockdown was confirmed by the real-time quantitative reverse transcription-polymerase chain reaction (QPCR) and ELISA.

### Real-Time QPCR

The QPCR assay was performed using Taqman Gene Expression Assays (Applied Biosystems, Foster City, CA, http://www.appliedbiosystems.com) as previously described [22, 23]. RNA was quantified with the *C*_t_ method, and glyceraldehyde 3-phosphate dehydrogenase (GAPDH) was used as a housekeeping gene [22, 23]. Taqman primers used were HIF-1α: Hs00153153_m1, HIF-2α: Hs01026149_m1, and p53: Hs00996818_m1. The Taqman primers for Oct-4, Nanog, ABCG2, and GAPDH are as previously described [23, 28].

### Cytoprotective Assays

For the in vitro cytoprotective assay, primary rat cardiomycotes were obtained from Cell Applications Inc. (San Diego, CA, http://www.cellapplications.com) [29] and grown in the rat cardiomyocyte growth medium as per manufacturer's instructions. SH-SY5Y neuroblastoma cells were obtained from ATCC. The cells were exposed to 24 hours of hypoxia followed by reoxygenation for next 24 hours, and the viability was measured by Trypan blue assay. The cell culture medium was replaced with hESCs CM 2 hours before exposing to hypoxia. The in vivo bone marrow (BM) stem cell cytoprotection experiments were carried out in BALB/c mice as described previously [14]. Briefly, 10–12-week-old BALB/c mice (Charles River Laboratories International, MA) were injected with hESCs CM i.p. 3 hours before the i.p. injection of high-dose 120 mg/kg carboplatin. On the day 5 of injection, mice were sacrificed, BMs were collected, and both the hematopoietic stem cell (HSC) and mesenchymal stem cell (MSC) fractions were isolated by using the easy-sep magnetic sorting kits (#19756 for HSC and #19771 for MSC; Stem Cell Technologies, BC, http://www.stemcell.com). The isolated cells were then subjected to colony forming unit (CFU) assay for HSCs and MSCs as described elsewhere [14].

### Limiting Dilution Assay for Teratoma Formation

The in vivo teratoma study was performed by injecting hESCs mixed with 1:2 Matrigel to non-obese diabetic severe combined immunodeficient mice subcutaneously as described [23]. The number of hESCs injected s.c. to mice were as follows: 2 × 10^6^ (5), 1 × 10^5^ (10), 1 × 10^4^ (10), 1 × 10^3^ (10), 1 × 10^1^ (10), and the number within parentheses representing number of animal used for the experiments. Mice were observed for 1–6 months for the teratoma formation. To confirm teratoma formation, the xenografts were fixed in formalin and subjected to H&E study [23]. In a separate experiment, a portion of Matrigel plugs were removed 3 weeks after implantation to mice as described [21] and frozen fixed to perform immunostaining [23] for Nanog expressing cells by using a anti-Nanog antibody (Supporting Information Table 1) [28].

### Statistical Analysis

The statistical analysis was performed using GraphPad Prism 4.0 (GraphPad Software Inc., CA, http://www.graphpad.com) using either Student *t* test or one-way analysis of variance with Newman-Keul post hoc test. The flow cytometry data were analyzed by flowjo (Tree Star, Inc., OR) [23]. The analysis of teratomagenic efficiency was done by extreme limiting dilution software [30] (http://bioinf.wehi.edu.au/software/elda)

## RESULTS

### P53 and HIF-2α Are Upregulated in hESCs Following Hypoxia/Oxidative Stress

**Figure 1:**
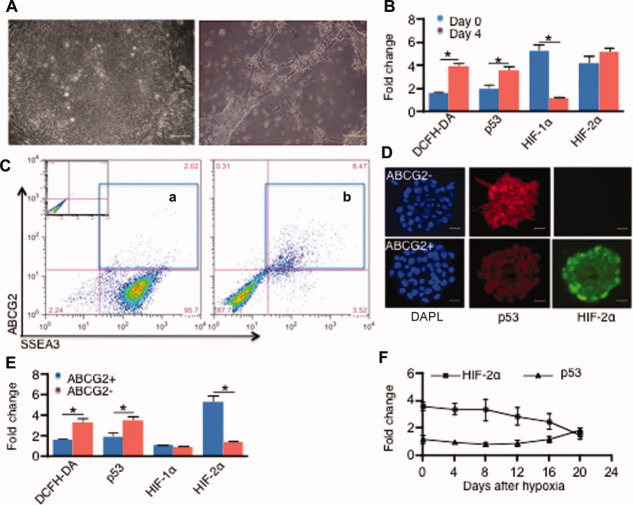
SSEA3+/ABCG2+ fraction survives and exhibits high HIF-2α and low p53 activity during exposure to hypoxia/reoxygenation. (A): Morphological evidence of BGO1 human embryonic stem cell differentiation and death (image to the right) following exposure to extreme hypoxia followed by 4 days of reoxygenation. The hypoxia- and reoxygenation-treated cells showed elongated neurite processes including fibroblastic-like features as well as evidence of cell fragmentation. (B): The fold change in reactive oxygen species (DCFH-DA), p53, and HIF-2α in the BGO1 cells is shown in (A), following day-4 post-hypoxia/reoxygenation. (C): Flow cytometry showing expansion (a vs. b compartments) of the SSEA3+/ABCG2+ fraction on the day-4 post-hypoxia/reoxygenation. Inset shows the profile of isotype control. (D): Immunofluorescence labeling of SSEA3+/ABCG2− and SSEA3+/ABCG2+ fractions on day-4 post-hypoxia/reoxygenation showing a high HIF-2α and low p53 signal in the SSEA3+/ABCG2+ fraction (DAPI, nuclear stain). (E): The corresponding fold change in DCFH-DA, p53, and HIFs in the SSEA3+/ABCG2+ versus SSEA3+/ABCG2− fractions on the day-4 post-hypoxia/reoxygenation. (F): Protein levels of HIF-2α and p53 in the SSEA3+/ABCG2+ fraction were measured by ELISA. Note high HIF-2α versus low p53 until day 20. *, *p* < .05; *n* = 4. Scale bar = 40 μm (A); 10 μm (D). Abbreviations: DAPI, 4′,6-diamidino-2-phenylindole; DCFH-DA, 2′,7′-dichlorfluorescein-diacetate; HIF, hypoxia inducible factor.

To investigate whether exposure to hypoxia followed by reoxygenation (i.e., oxidative stress) activates p53 as well as HIF-2α in ESCs, BGO1, human ESCs were exposed to extreme hypoxia (*p*O_2_ ∼ 0.1% for 24 hours) followed by reoxygenation (Supporting Information Fig. 1a) [22], and changes in ROS, p53, and HIFs were measured. We found that BGO1 cells underwent marked morphological changes of differentiation and cell death (Fig. 1A) with an accompanying fourfold increase in ROS level (Fig. 1B). While the p53 protein level was increased fourfold by day 4 (Fig. 1B), HIF-2α protein was already rapidly elevated threefold after 1 day post-hypoxia (not shown) and remained sustained during the day-4 reoxygenation period (Fig. 1B). In contrast, HIF-1α protein expression increased immediately during hypoxia (day 0) and then showed a rapid reduction in expression by day-4 post-hypoxia (Fig. 1B). These results suggest that activation of both p53 and HIF-2α is sustained on day 4 during the reoxygenation phase following hypoxia. In contrast, HIF-1α decreased during the reoxygenation phase, which is consistent with the previous findings that HIF-1α mRNA is unstable whereas HIF-2α mRNA could be sustained for a prolonged period after hypoxia [6].

### The High HIF-2α and Low p53 Cells Are Enriched in the SSEA3+/ABCG2+ Fraction

We next considered that the high HIF-2α cell fraction might be enriched within the undifferentiated ESC fraction and would express ABCG2, since this is one of the target genes of HIF-2α [8]. To investigate this possibility, we first evaluated whether the high HIF-2α expressing hESCs on day-0 and day-4 post-hypoxia treatment overlapped with ABCG2+ expression. We found that most of the HIF-2α expressing cells were positive for ABCG2 (Supporting Information Fig. 1b). Second, we sorted by flow cytometry a surviving (propidium iodide negative) undifferentiated SSEA3+ fraction expressing ABCG2. The flow sorting showed that only 12% ± 3% (*p* < .05; *n* = 4) SSEA3+ cells survived by day-4 post-hypoxia/reoxygenation (Fig. 1C), which was a eightfold reduction in the SSEA3+ fraction (*p* < .05; *n* = 4). We then found that the ABCG2+/SSEA3+ fraction (Fig. 1C) increased by fourfold (Fig. 1C; 4.2 ± 1.1, *p* < .05; *n* = 4) by day-4 post-hypoxia/reoxygenation.

Third, we compared the HIF-2α protein levels as well as transcriptional activities between the SSEA3+/ABCG2+ and SSEA3+/ABCG2− fractions using several assays. Immunofluorescence labeling revealed a strong expression of HIF-2α in the ABCG2+ fraction versus the ABCG2− fraction (Fig. 1D), which was confirmed at the protein level as measured by ELISA (Fig. 1E), and at gene expression and the transcriptional activity level (Supporting Information Fig. 2a, 2b). The HIF-1α expression was not sustained in the day-4 post-hypoxia/reoxygenation ABCG2+ fraction (Fig. 1E; Supporting Information Fig. 2a).

Taken together, our results suggest that HIF-2α expression was enriched in the SSEA3+/ABCG2+ fraction as compared to the SSEA3+/ABCG2− fraction.

Next we compared the protein level and transcriptional activity of p53 in SSEA3+/ABCG2+ versus SSEA3+/ABCG2−. Immunofluorescence labeling indicated a low expression of p53 in the ABCG2+ fraction versus the ABCG2− fraction (Fig. 1D), which was also confirmed quantitatively by ELISA (Fig. 1E), and by transcriptional activity as measured with the transcriptional filter assay (Supporting Information Fig. 2b). These results suggest that the SSEA3+/ABCG2+ fraction expresses a low level of p53 as compared to the SSEA3+/ABCG2− fraction.

We reasoned that a high HIF-2α and low p53 activity could be associated with higher survival and less DNA damage in the SSEA3+/ABCG2+ fraction versus the SSEA3+/ABCG2− fraction. Hence we measured levels of ROS, 8-OHdG, and caspase-3 and found these to be four- to fivefold higher in the SSEA3+/ABCG2− versus the SSEA3+/ABCG2+ fraction (Supporting Information Fig. 2c). The trypan blue viability assay showed a 3.5-fold higher survival of SSEA3+/ABCG2+ versus SSEA3+/ABCG2− (*p* < .05; *n* = 4; data not shown). Thus, we conclude that the SSEA3+/ABCG2+ fraction was characterized by higher survival and less DNA damage as compared to the SSEA3+/ABCG2− fraction.

We then investigated whether the high HIF-2α/low p53/ABCG2+ phenotype generated through hypoxia/reoxygenation hereon designated ABCG2+hox could persist in vitro for a prolonged period of time. We found that this cell fraction actively proliferated in the ESC medium for several weeks (three passages; total of 25 days). During this period, p53 and HIF-2α levels were measured by ELISA. The protein levels were compared with day-0 post-hypoxia/reoxygenation ABCG2+ cell fraction. We found that the post-hypoxia/reoxygenation ABCG2+hox fraction showed a sustained phenotype of low p53 and high HIF-2α expressions that lasted for 20 days (Fig. 1F).

Taken together, we conclude that hypoxia/reoxygenation endows a subfraction of BGO1 ESCs with enhanced survival and is associated with high HIF-2α and low p53 activities. This high HIF-2α and low p53 phenotype could persist in vitro for more than 2 weeks and was mainly confined to the ABCG2+ fraction.

### ABCG2+Hox Cells Exhibit a Highly Undifferentiated and Cytoprotective State

**Figure 2:**
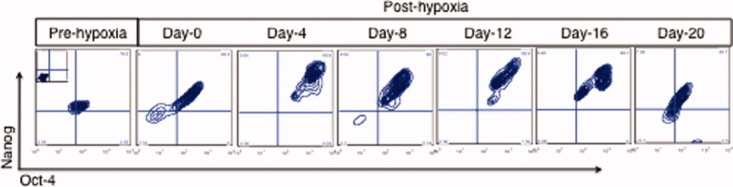
A representative flow cytometry profile depicting the sustained expression of high Nanog and Oct-4 in the BGO1 ABCG2+hox cells following exposure to hypoxia/reoxygenation. The ABCG2+hox cells were flow cytometry sorted as shown in Figure 1D and maintained in the human embryonic stem cells (hESCs) culture media for next 3 weeks. In specific interval of time, the ESCs were fixed and subjected to flow cytometry expression of Nanog and Oct-4 as described [23]. The inset panel at the top of the prehypoxia panel indicates the isotype control. The BGO1 hESCs were fixed and permeabilized before subjecting to incubation with primary and secondary (Alexa 488 for Oct-4 and phycoerythrin for Nanog) antibodies. Results shown are representative of a typical experiment (*n* = 4; data from 5,000 single-cell events).

**Figure 3:**
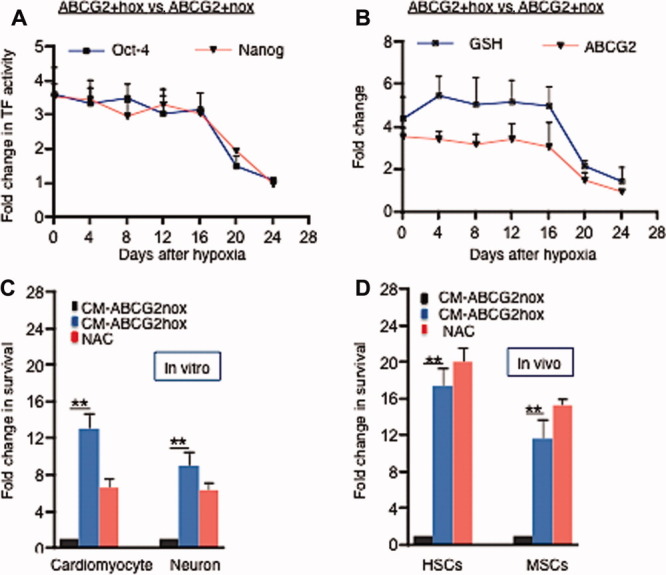
ABCG2+hox fraction exhibits a highly undifferentiated and cytoprotective state. (A): The fold change in the transcriptional activity of Oct-4 and Nanog in ABCG2+hox relative to ABCG2+nox cells. (B): The fold change of the GSH and ABCG2 levels in the ABCG2+hox relative to ABCG2+nox cells. The GSH level was measured in the CM obtained from the cells. The ABCG2 level was measured by ELISA. (C): Fold change in cell survival, measured by trypan blue exclusion, of human primary cardiomyocytes and neuronal-like cells (SHSY5Y N-type neuroblastoma cell line) [31] following exposure to conditioned medium obtained from human embryonic stem cells versus nonexposed medium. The antioxidant NAC was used as a positive control. (D): Fold change in the survival of the mouse HSCs and MSCs following treatment of carboplatin-treated mice with CM obtained from ABCG2+hox versus ABCG2+nox cells. *, *p* < .05; **, *p* < .001; A, B, C *n* = 4, and D, *n* = 3. Abbreviations: CM, conditioned media; GSH, glutathione; HSC, hematopoietic stem cell; MSC, mesenchymal stem cell; NAC, *N*-acetyl cysteine; TF, transcription factor.

To investigate whether the ABCG+2hox fraction maintained an undifferentiated stemness state throughout the 3 week culture period of low p53/high HIF-2α expression, we measured the flow cytometry expression and transcriptional activity of Oct-4 and Nanog in the ABCG2+hox fraction and compared it with ABCG2+nox (designating the SSEA3+/ABCG2+ fraction maintained under normoxia and not exposed to hypoxia/reoxygenation). First, we found that the levels of Oct-4 and Nanog protein expression measured by flow cytometry were sustained in ABCG2+hox over the 2 week period (Fig. 2). Second, the transcriptional activity of Oct-4 and Nanog were sustained three- to fourfold higher in ABCG2+hox versus ABCG2+nox over the 2 week period (Fig. 3A). Thereafter the transcriptional activity level returned to a baseline level corresponding to the transcriptional activities of Oct-4 and Nanog in the ABCG2+nox fraction by day-20 of reoxygenation (Fig. 3A), and similar to the period of high HIF-2α/lowp53 state of ABCG2+hox as previously described in Figure 1F. This result suggested that the ABCG2+hox fraction, having a sustained high HIF-2α activity, also sustained a higher Nanog and Oct-4 transcriptional activity over the 2 week culture period.

Consequently, we speculated that the ABCG2 protein level might also be sustained at a higher level in the ABCG2+hox fraction for a correspondingly similar period. Previous studies have shown that the ABCG2 efflux pump confers a cytoprotective effect on ESCs by reducing intracellular ROS [9]. A recent study reported that ABCG2 also secretes GSH, a potent cellular antioxidant, into the extracellular space [13]. We, and others, reported that GSH exerts a cytoprotective activity on mammalian cells, including BM stem cells, against hypoxia/reperfusion as well as cancer chemotherapy-induced toxicities [14]. Hence, a corresponding increase in GSH secretion by the ABCG2+hox fraction might confer cytoprotection on nearby cells exposed to ROS-induced toxicity. We investigated these possibilities as follows.

First, we measured the ABCG2 protein level in the ABCG2+hox versus ABCG2+nox fractions by ELISA assay. We found a sustained threefold increase of ABCG2 expression in the ABCG2+hox versus the ABCG+2nox fraction (Fig. 3B). Second, we measured GSH secretion in the CM obtained from the ABCG2+hox and ABCG2+nox fractions with a GSH quantitative assay that is very sensitive for measurement of GSH in stem cells [14]. We found a fivefold increase in the GSH level in the CM obtained from the ABCG2+hox fraction (Fig. 3B). We also noted that the level of ABCG2 and GSH returned to the baseline level on day 20 corresponding to the return of the high-HIF-2α/low p53 state to its baseline state as in the ABCG2+nox phenotype (Fig. 3B). Thus, it appears that the ABCG2+hox phenotype is associated with high GSH secretion into the CM.

Third, we compared the in vitro cytoprotection potential of the ABCG2+hox- versus ABCG2+nox-derived CM. CM was collected from 1 × 10^6^ ABCG2+hox cells in 4 ml, on day-8 post-hypoxia, and used to treat cardiomyocytes and neuronal cells (SH-SY5Y cell line [31]) exposed to hypoxia/reperfusion, and was compared to nonexposed medium as a control. We then assessed the effect on survival and found a 10–12-fold increase in the survival of the cardiomyocytes and neuronal cells when treated with the CM of ABCG2+hox versus CM from the ABCG2+nox fraction (Fig. 3C). Thus we demonstrated that the ABCG2+hox fraction CM exerted a significant cytoprotective activity on differentiated cardiomyocytes and neuronal cells, suggesting a mechanism whereby the ABCG2+hox fraction is cytoprotective.

To obtain in vivo evidence of the cytoprotective activity of the ABCG2+hox-derived CM, we used a murine model of carboplatin-induced BM stem cell toxicity assay developed in our laboratory [14]. In this assay, antioxidants were shown to protect BM HSCs and MSCs from high-dose carboplatin-induced toxicity [14]. We pretreated carboplatin-exposed mice with CM derived from ABCG2+hox compared to CM from ABCG2+nox. The CM were collected from 1 × 10^6^ ABCG2+hox and ABCG2+nox cells in 2 ml, on day-8 post-hypoxia, and 1 ml was injected i.p. to mice. We then measured both HSC and MSC survival using established assays [14]. The CM was compared to *N*-acetyl-cysteine (NAC) a potent antioxidant, the latter having been shown to cytoprotect stem cells [32–34]. We found that the CM derived from the ABCG2+hox cells showed a 17-fold cytoprotection of HSC and 10-fold protection of MSCs (Fig. 3D). Interestingly, this cytoprotective activity in CM was roughly equivalent to NAC. Thus, we conclude that the ABCG2+hox CM showed potent cytoprotective activity in vivo against BM toxicity induced by carboplatin, a commonly used anticancer agent in clinics. Taken together, our observations above suggest that the ABCG2+hox fraction attains a highly undifferentiated state (henceforth known as enhanced stemness state) and exhibits potent cytoprotective activity.

Considering the present interest in the potential use of hESCs for tissue regeneration, the results obtained from the BGO1 CM are provocative. To evaluate whether CM derived from the ABCG2+hox fraction of other hESC lines could also exhibit similar regenerative potential, we repeated the above experiments with the H9 hESC line and obtained similar results. These results are described in Supporting Information Figure 3 and suggest that the H9 ABCG2+ hox cells exhibited a high HIF-2α, Oct-4, Nanog and low p53 phenotype, and exhibited in vivo cytoprotective activity comparable to that of NAC (Supporting Information Fig. 3). Thus, we conclude that the ABCG2+ hox fraction in both the BGO1 and H9 hESCs exhibited cytoprotective activities. We then decided to evaluate the characteristic of the ABCG2+hox phenotype using the BGO1-derived ABCG2+hox cells.

### The Enhanced Stemness State Portends an Extreme Degree of Self-Sufficiency

We noted that the ABCG2+hox fraction showed a higher level of Nanog activity (Figs. 2, 3A). Since very high Nanog expression leads to an embryonal carcinomatous (EC) like state of extreme self-sufficiency [16, 17, 35], we investigated whether the ABCG2+hox fraction attained extreme self-sufficiency.

**Figure 4:**
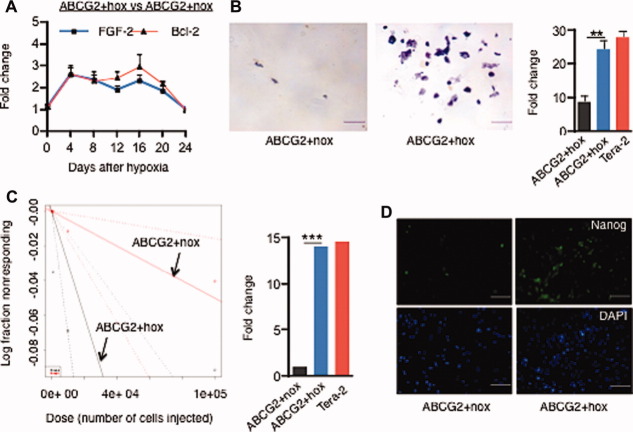
ABCG2+hox fraction exhibits a phenotype of self-sufficiency. (A): The fold change in FGF-2 and Bcl-2 in ABCG2+hox cells relative to ABCG2+nox cells. (B): The single-cell clonogenic assay showing the alkaline phosphatase staining of abundant colony formation in the ABCG2+hox cell fraction. The histogram shows the significant fold increase in colonies derived from the ABCG2+hox fraction. Tera-2-derived colonies were used as a control. (C): The in vivo limiting dilution assay showing higher teratomagenic formation efficiency of ABCG2+hox versus ABCG2+nox. Tera-2 teratoma formation was used as a control. (D): Immunofluorescence staining on a frozen section of ABCG2+ cells containing Matrigel plug showing expression of Nanog positive cells. The quantification of the Nanog expressing cells is given in the Supporting Information Figure 6. *, *p* < .05; **, *p* < .001; ***, *p* < .00001, *n* = 4. Scale bar = 40 μm (B) and (D). Abbreviations: DAPI, 4′,6-diamidino-2-phenylindole; FGF-2, fibroblast growth factor-2.

First, we assayed for the expression of FGF-2 and Bcl-2, involved in growth and regulation of apoptosis and associated with extreme self-sufficiency of ESCs and EC cells [36]. We found that FGF-2 and Bcl-2 expressions were upregulated two- to threefold in the ABCG2+hox versus ABCG2+nox fractions (Fig. 4A). Second, we performed a standard clonogenic assay, minus supplementation with a ROCK inhibitor [37], on flow-sorted ABCG2+hox and ABCG2+nox cells grown on Matrigel as single cells, and the ESC colony formation was measured after 4 days. We found that the ABCG2+hox cells showed a 10-fold higher colony forming capacity than the ABCG2+nox cells (Fig. 4B). The colony forming efficiency was similar to that of Tera-2 cells (Fig. 4B). Our results are consistent with previous reports that high Nanog and Bcl-2 levels enhance the ability of flow-sorted single hESC to survive without the addition of the ROCK inhibitor [36]. Thus, we suggest that the ABCG2+hox cells demonstrated a significantly greater degree of self-sufficiency than the ABCG2+nox fraction in the in vitro clonogenic assay.

Next, we performed the in vivo teratoma assay to further characterize the enhanced state of self-sufficiency. The ABCG2+hox and ABCG2+nox were injected subcutaneously into immunocompromised mice [38] to obtain teratomas (Supporting Information Fig. 4), and a serial dilution assay was performed to compare the teratomagenic frequency between the ABCG2+hox and ABCG2+nox. Tera-2 was used as a control. We found a 15-fold increase in teratomagenic efficiency of ABCG2+hox versus ABCG2+nox (Fig. 4C) and comparable to the Tera-2 cells (Fig. 4C). We noted that the ABCG2+hox fraction contained a eightfold higher level of Nanog expression (Fig. 4D, and detail quantification given in the Supporting Information Fig. 5) than the ABCG2+nox fraction in the Matrigel plug, 10 days after implantation. This is expected, since higher self-sufficiency would contribute to higher cell survival and resistance to differentiation of ESCs during subcutaneous implantation. We noted that a small fraction of the ABCG2+ nox cells in the Matrigel plug continued to express high Nanog (Fig. 4D; Supporting Information Fig. 5). Considering that the Matrigel plug microenvironment is hypoxic [22, 23], we speculate that a few of the ABCG2+nox cells in the Matrigel plugs might have undergone transient reprogramming to acquire ABCG2+hox phenotype. We also noted that the ABCG2+hox xenograft-derived cells did not form secondary teratomas suggesting that the ABCG2+hox fraction exhibited EC-like characteristics of self-sufficiency for a transient period of time in vivo but did not exhibit the full-blown EC-like malignant phenotype of teratocarcinoma as does Tera-2.

Taken together, we concluded that the ABCG2+hox fraction underwent a temporary but measurable phenotypic switch to an enhanced stemness state of extreme self-sufficiency but did not exhibit malignant transformation to teratocarcinoma.

### HIF-2α-Mediated p53 Suppression Maintained the Enhanced Stemness State

Because the pluripotency state is stabilized in a metastable state, wherein Nanog is maintained within a threshold level [39], the high Nanog expression state is unstable and therefore returns to a basal level within hours to days [40, 41]. Multiple mechanisms, including p53 activity are likely involved in maintenance of a balanced state of Nanog expression [39]. We investigated the molecular mechanism of how the ABCG2+hox fraction maintained a state of high Nanog and associated self-sufficiency for a prolonged period of time without returning to the basal state of pluripotency. We speculated that high HIF-2α might suppress p53 activity to allow the high Nanog state to transiently retain stability and tested this idea as follows.

First, we investigated the phenotypic switching of ABCG2+nox to ABCG2+hox cells when they were made deficient in HIF-2α and HIF-1α. Both HIF-1α and HIF-2α were downregulated in BGO1 cells by siRNA gene silencing (Supporting Information Fig. 6a) and also by treatment with FM19G11 (Calbiochem), an inhibitor of HIFs [21]. HIF-deficient BGO1 cells were then exposed to hypoxia/reoxygenation to obtain the ABCG2+hox fraction. The inhibition of HIF-2α by siRNA as well as FM19G11 led to an eight- to ninefold reduction of the ABCG2+hox fraction (Supporting Information Fig. 6b). In contrast, the inhibition of HIF-1α by siRNA gene silencing of HIF-1α did not affect the ABCG2+hox fraction (Supporting Information Fig. 6b). These results suggest that HIF-2α and not HIF-1α was required for the survival and/or expansion of the ABCG2+hox fraction during hypoxia/reoxygenation.

Next, we investigated the potential HIF-2α-mediated suppression of p53 by treating the ABCG2+hox fraction with the HIF-2α inhibitor FM19G11 or siRNA HIF-2α and then determining cell survival and the expressions of p53. We found that HIF-2α inhibition resulted in a fourfold increase in apoptosis as measured by caspase-3 activity [22] (Fig. 5A) and corresponding increase in p53 expression (Fig. 5B). The enhanced stemness state Nanog and ABCG2 expressions were downregulated (Fig. 5C). These results suggested that downregulating HIF-2α led to increased expression of p53 and associated differentiation of the enhanced stemness state.

**5:**
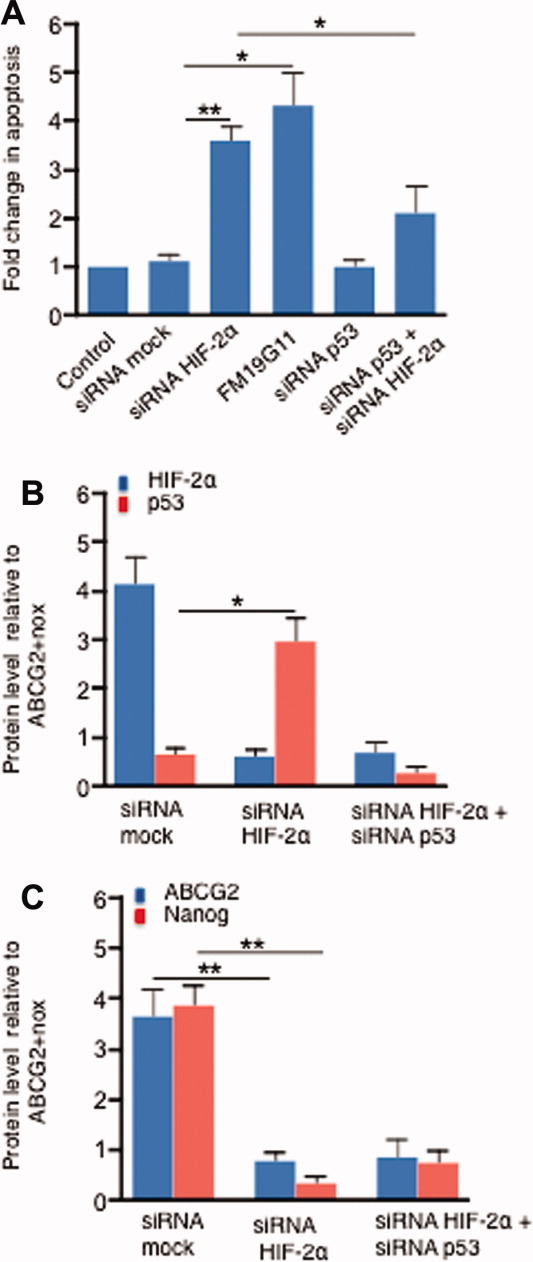
HIF-2α is involved in the maintenance of the enhanced stemness state. (A): The fold change in the apoptosis (caspase-3 activity) of the ABCG2+hox (day-8 post-hypoxia) cells versus ABCG2+nox cells following treatment with various inhibitors of HIF-2α and p53. (B): The fold change of HIF-2α and p53 protein levels in the surviving cells of (A). (C): The fold change in Oct-4 and ABCG2 protein levels in the surviving cells of (A).*, *p* < .05; **, *p* < .001; *n* = 3. Abbreviations: HIF, hypoxia inducible factor; siRNA, small interfering RNA.

We interrogated whether the increased p53 activity was associated with increased apoptosis following HIF-2α inhibition. First, the HIF-2α inhibitor-treated ABCG2+hox fraction was treated with either siRNA p53 or pifithrin-α, a p53 inhibitor [42]. We found that siRNA gene silencing of p53 enhanced survival of the ABCG2+hox fraction (Fig. 5A). A similar result was obtained with pifithrin-α treatment (data not shown). These results suggested that HIF-2α was involved in the inhibition of p53-mediated apoptosis/differentiation of the ABCG2+hox fraction. We concluded that high HIF-2α suppressed the p53 activity to enhance the survival of ABCG2+hox cells.

### HIF-2α Modulates the p53/MDM2 Oscillation System

Because, p53 is associated with p53/MDM2 oscillation having a period of high MDM2 and low p53 state [43–45], we evaluated the potential role of HIF-2α in altering the oscillation to sustain a high MDM2 and low p53 state in the ABCG2+hox cells.

First, the ABCG2+hox cells were treated with Nutlin-3, an MDM2 inhibitor, and the survival as well as differentiation of the treated cells were evaluated by performing appropriate assays. We noted that Nutlin-3 inhibition led to a ninefold reduction in the ABCG2+hox fraction (Supporting Information Fig. 6b). Next, we observed a four- to fivefold reduction in Nanog, Oct-4, and ABCG2 levels (*p* < .05; data not shown). These results suggest that a high MDM2 state was associated with the enhanced stemness state.

**Figure 6:**
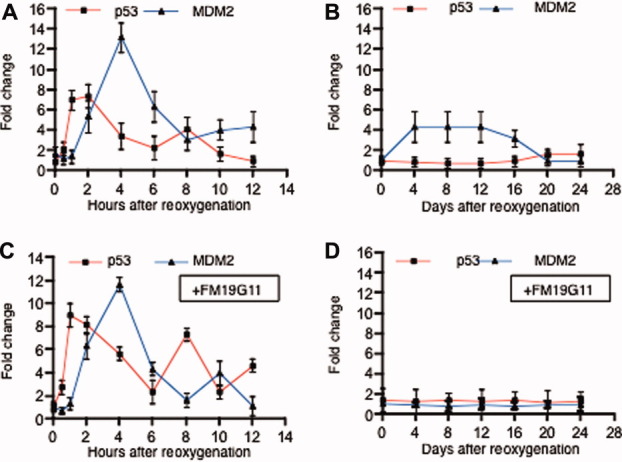
HIF-2α is involved in the generation of an altered p53/MDM2 oscillation in the ABCG2+hox. (A): The kinetic changes of p53 and MDM2 proteins in ABCG2+ hox cells immediately, (B) days after, and (C) and (D) following treatment with hypoxia inducible factor (HIF) inhibitor FM19G11 [21]. The p53 and MDM2 levels were checked by InCell western and the induction fold of p53 and MDM2 after reoxygenation was calculated at each time point. Data presented represent the mean of four experiments. Similar results of fold induction of p53 and MDM2 was obtained by ELISA measurement (described in text).

Next, we characterized the effect of HIF-2α inhibition in the p53/MDM2 oscillation state of ABCG2+hox cells by measuring the protein levels of p53 and MDM2 by InCell western and ELISA over a period of 3 weeks. The results obtained by performing InCell western are shown in Figure 6. We found a p53/MDM2 oscillation pattern in the ABCG2+hox within the first 12 hours of hypoxia (Fig. 6A). The oscillation then ceased, but a high MDM2 and low p53 state persisted for 2 weeks (Fig. 6B). We found similar evidence of the p53/MDM2 oscillation by the ELISA measurement (data not shown). Most importantly, HIF-2α inhibition by FM19G11 led to a p53/MDM2 oscillation of high p53 amplitude, and low MDM2 state (Fig. 6C), and then return of both p53 and MDM2 levels to their basal state (Fig. 6D). We noted that the p53/MDM2 oscillation in the SSEA3+/ABCG2− fraction, that did not show high HIF-2α, also did not exhibit a high MDM2 state (Supporting Information Fig. 7). These results suggest that BGO1-derived ABCG2+hox exhibited an abnormal state of p53/MDM2 oscillation characterized by a sustained high MDM2 state that lasted for more than 2 weeks. Treatment with HIF inhibitor FM19G11 prevented the occurrence of this sustained high MDM2 state.

To further investigate the role of HIF-2α in the initiation of the abnormal high MDM2 state of the p53/MDM2 oscillation, we performed siRNA HIF-2α silencing of BGO1 before subjecting cells to hypoxia/reoxygenation as described in Supporting Information Figure 7. The post-hypoxia ABCG2+hox was analyzed by ELISA assay to obtain p53 and MDM2 protein levels. We also performed the experiment in H9 hESCs that showed the presence of an ABCG2+hox phenotype (Supporting Information Fig. 3). The result obtained with BGO1 was similar to data described in Figure 6D. The findings on H9 are described in Supporting Information Figure 7c, 7d. First, we noted that H9-derived ABCG2+hox showed a similar pattern of an abnormal state of low p53 and high MDM2 that was sustained for 2 weeks. Second, siRNA HIF-2α treatment lowered the MDM2 level to a basal state in H9 (Supporting Information Fig. 7d). We concluded that the hypoxia-induced HIF-2α was associated with the transient stabilization of a high MDM2 and low p53 state in the ABCG2+ hox fraction of both BGO1 and H9 hESCs.

## DISCUSSION

hESCs are pluripotent stem cells derived from inner cell mass of developing embryos and have adapted to specialized in vitro culture conditions to maintain an undifferentiated state with self-renewal capacity (stemness state). We now report that a rare fraction of ESCs, the ABCG2+hox fraction, undergoes transition under hypoxia/reoxygenation into a transient, but reasonably persistent phenotype of high HIF-2α, Nanog, Oct4, and ABCG2 activity. The phenotype was associated with an abnormal p53 and MDM2 oscillation characterized by low p53 and high MDM2 state. This phenotype was correlated with a state of enhanced stemness that was permissive for survival and self-sufficiency. Importantly, in the context of tissue repair and regeneration, the enhanced stemness state is characterized by high cytoprotective activity. Whether similar activity could be attributed to adult stem cells are now under active investigation.

Thus, we propose that hESCs use a novel mechanism of enhanced stemness reprogramming to acquire a higher degree of functionality for regeneration, where HIF-2α, turned on by hypoxia and oxidative stress, sustains functional activity for at least 2 weeks after the initial induction.

Although generalization of the enhanced stemness reprogramming as a defense strategy common to stem cells would be highly speculative, we suggest that if used, this potential strategy of cytoprotection (altruism) might benefit hESCs as well as adult stem cells to survive and maintain stemness and regenerative potential at the site of injury.

Sites of tissue injury are subjected to cycles of hypoxia/reoxygenation during the repair process. These sites appear to attract stem cells whose roles are still poorly understood although there is a growing understanding that stem cells may assist in the repair process either directly or through production of critical factors [1, 46]. Here we subjected hESCs to hypoxia/reoxygenation mimicking the site of injury. The hESC model has the advantage that distinct phenotypes can be discriminated in vitro and in vivo and the factors that maintain the stem cell state have been well elaborated [16, 47].

The time frame of this hESC cell phenotype of enhanced stemness state, characterized by higher degree of self-sufficiency and cytoprotective ability, corresponds to prolonged and abnormal state of p53/MDM2 oscillation. This altered state of the oscillation system was associated with a high HIF-2α activity since inhibiting HIF-2α led to phenotypic differentiation of the enhanced stemness phenotype. Thus the HIF-2α and p53 systems, coordinately upregulated during hypoxia and oxidative stress, could contribute to the emergence of a rare fraction of hESCs harboring high HIF-2α and low p53, that is, a state of enhanced stemness state of self-sufficiency.

This transient state now permitted hESCs to acquire a highly cytoprotective, regenerative activity. This cytoprotective activity appears to be mediated by a high level of functional ABCG2, an efflux pump that secretes GSH, a potent endogenous antioxidant. We showed that the enhanced stemness state was associated with a high level of GSH secretion, with GSH secreted into the CM, which was capable of cytoprotecting cardiomyocytes and primitive neuronal cells represented by SH-SY5Y cell line from the toxic effect of hypoxia/reperfusion injury in vitro as well as chemotherapeutic induced BM cell cytotoxicity in vivo. Whether other factors in the CM act cooperatively with GSH remains to be determined, however, these observations strongly support the notion that stem cells in sites of injury and repair condition the environment and may contribute effectively to the regenerative process.

The suppression of p53 was required to initiate and maintain the enhance stemness state, and therefore, the cytoprotective activity. However, one potential trade-off of p53 suppression would be the imbalance in the stemness state that could lead to malignant transformation, a major concern of current stem cell-based therapies [17]. In this regard, we noted the observation that MDM2 is highly expressed in EC and positively regulates FGF-2 [48, 49]. Although we found that the ABCG2+hox cells were highly teratomagenic, ABCG2+hox cells did not exhibit teratocarcinoma activity, suggesting that the enhanced stemness state did not lead to malignant transformation. One reason may be that the transient nature of the enhanced stemness state functions as a possible safety mechanism that is built into stem cells to mitigate against malignancy. In this context, it would be of crucial importance to determine how this safety mechanism is overridden, for example, by an oncogenic event, in the switch to a malignant phenotype. Our model might be ideally suited to probe this vital question.

## CONCLUSION

In summary, we now propose a novel mechanism whereby hESCs might contribute to healing when exposed to an extreme oxidative stress state of hypoxia/reoxygenation. In this potential mechanism, a fraction of highly undifferentiated stem cells acquire a transient state of enhanced stemness reprogramming characterized by high antioxidants and their secretion, where p53 is temporarily downregulated. Thus in this model, and perhaps counterintuitive, a few stem cells can acquire a higher degree of self-sufficiency to resist differentiation in the hypoxia/oxidative stress microenvironment to protect other cells from oxidative stress damage. Is this enhanced stemness reprogramming then a form of cell altruism [50] that could benefit the organism from the earliest developmental beginnings?

## Supporting Information

Supplementary Figure 1 (a) A schematic representation of the experimental design of hypoxia/reoxygenation. (b) A representative (n = 4) flow cytometry profile of post-hypoxia BGO1 cells depicting sustained expression of HIF-2α mainly confirmed to the ABCG2+ fraction on day-4 of reoxygenation. The BGO1 hESCs cells were fixed, and stained with both ABCG2 and HIF-2α antibodies, and then counterstained with FITC and PE secondary antibodies respectively. The insert on the top of the far-left flow cytometry profile represents the isotype control. (n = 4; data from 5,000 single-cell events). * hESCs cultures were allowed to grow on average 5-7 days resulting in colonies as depicited Figure 1A (main text).

Supplementary Figure 2 (a) Real time PCR (QPCR) data showing the expression of p53, HIF-2α, and HIF-2α expression in the SSEA3+/ABCG2+ and SSEA3+/ABCG2− fractions of BGO1 hESCs between pre-hypoxia and day-4 post hypoxia. QPCR data was first normalized to GAPDH value and then analyzed using – CT method as described^22,23^ (b) The SSEA3+/ABCG2+ fraction shows high HIF-2α and low p53 activity as measured by the transcriptional activity assay. (c) The ABCG2− versus ABCG2+ fraction of SSEA3+ hESCs showed higher ROS generation, Oxidative DNA damage, and Caspase-3 activity. The BGO1 hESCs cells were exposed to hypoxia, harvested on day-4 re-oxygenation and the values were expressed as the fold change between day-4 in hypoxia. The *P* < 0.001; n = 3

Supplementary Figure 3 H9 derived ABCG2+ hox fraction exhibits highly undifferentiated and cytoprotective state a&b. The fold change in the transcriptional activity of HIF-2α, p53, Oct-4 and Nanog in ABCG2+hox relative to ABCG2+nox cells. c. Fold change in the survival of the mouse HSCs (CD34+ fraction) and MSCs (CD271+ fraction) following treatment of carboplatin-treated mice with conditioned media (CM) derived from the ABCG2+hox versus ABCG2+nox. *p<0.05, **p<0.001, ***<0.0001, n = 3

Supplementary Figure 4 In vivo pluripotency of ABCG2+hox cells. The right panel shows an enlarged area of the left panel (Square box) depicting immature cartilage (Red arrow), and gastrointestinal glands (Yellow arrow). ABCG2+hox cells (1x104 cells) were injected to NOD/SCID mice subcutaneously as described^23^, and a representative 1.5 cm size teratoma was subjected to histological examination by performing H&E staining.

Supplementary Figure 5 Representative flow cytometry images that quantify results presented in Figure 4d, main text. The 10 day old subcutaneous matrigel plugs (n = 5) containing BGO1 hESCs derived SSEA3+/ABCG2+ cells [obtained either before hypoxia (ABCG2+nox) or after day-4 post-hypoxia (ABCG2+hox)] were subjected to enzymatic dissociation 22 and staining with antibody against Nanog28 followed by flow cytometry (5,000 single-cell events were collected). Whereas the ABCG2+nox matrigel plugs contained 3.1 ± 0.5 percentage of Nanog positive cells, the ABCG2+hox matrigel plugs contained 23.5 ± 1.2 percentage of Nanog positive cells (n = 5, *p<0.0001).

Supplementary Figure 6 (a) QPCR and ELISA data showing the silencing of HIF-2α and p53 by Accell siRNA in ABCG2+hox. The viability tested by Trypan blue showed no significant toxicity following either with targeted (T) or non-targeted (NT) siRNA treatment. The HIF-2α and p53 silencing were assessed by QPCR and ELISA respectively 72-hours after the addition of Accell siRNA 1–M to ABCG2+hox cells (day-4 post hypoxia). The data was compared to the untreated cells. QPCR data was first normalized to GAPDH value and then analyzed using — CT method as described^22,23^ (b) Fold reduction of post-hypoxia (day 4) ABCG2+ hox cells following siRNA HIF-2α or FM19G11 (0.5–M/ 48 hours) or Nutlin-3 (10–M/48 hours) treatment compared to untreated BGO1 cells. The BGO1 cells treated with above mentioned siRNA or inhibitors were exposed to hypoxia, and the percentage of ABCG2+hox was evaluated by flow cytometry on day-4 post hypoxia. Note that siRNA HIF--α silencing showed no detectable changes in the percentage of ABCG2+hox. n=3. **p<0.001. We achieved HIF-α silencing of >80% protein level by ELISA (not shown).

Supplementary Figure 7 (a) The kinetic changes of the levels of p53 and MDM2 proteins (as measured by InCell western) depicted as induction fold in the SSEA3+/ABCG2− fraction immediately and (b) days after hypoxia/reoxygenation. (c) The kinetic changes of the levels of p53 and MDM2 proteins (as measured by ELISA) depicted as induction fold in the hESC line H9 derived SSEA3+/ABCG2+ fraction days after exposure to hypoxia, and (d) represent the state of p53/MDM2 kinetic changes of (c) when the siRNA HIF-2α treated H9 cells were exposed to hypoxia. Data presented represent the mean of four experiments (a&b) and three experiments (c & d).
