# Supplementary figures and images for: HIF-2α Suppresses p53 to Enhance the Stemness and Regenerative Potential of Human Embryonic Stem Cells
Source: Stem Cells. 2012 Aug;30(8):1685–95. doi: 10.1002/stem.1142 (PMC3584519; doi:10.1002/stem.1142)

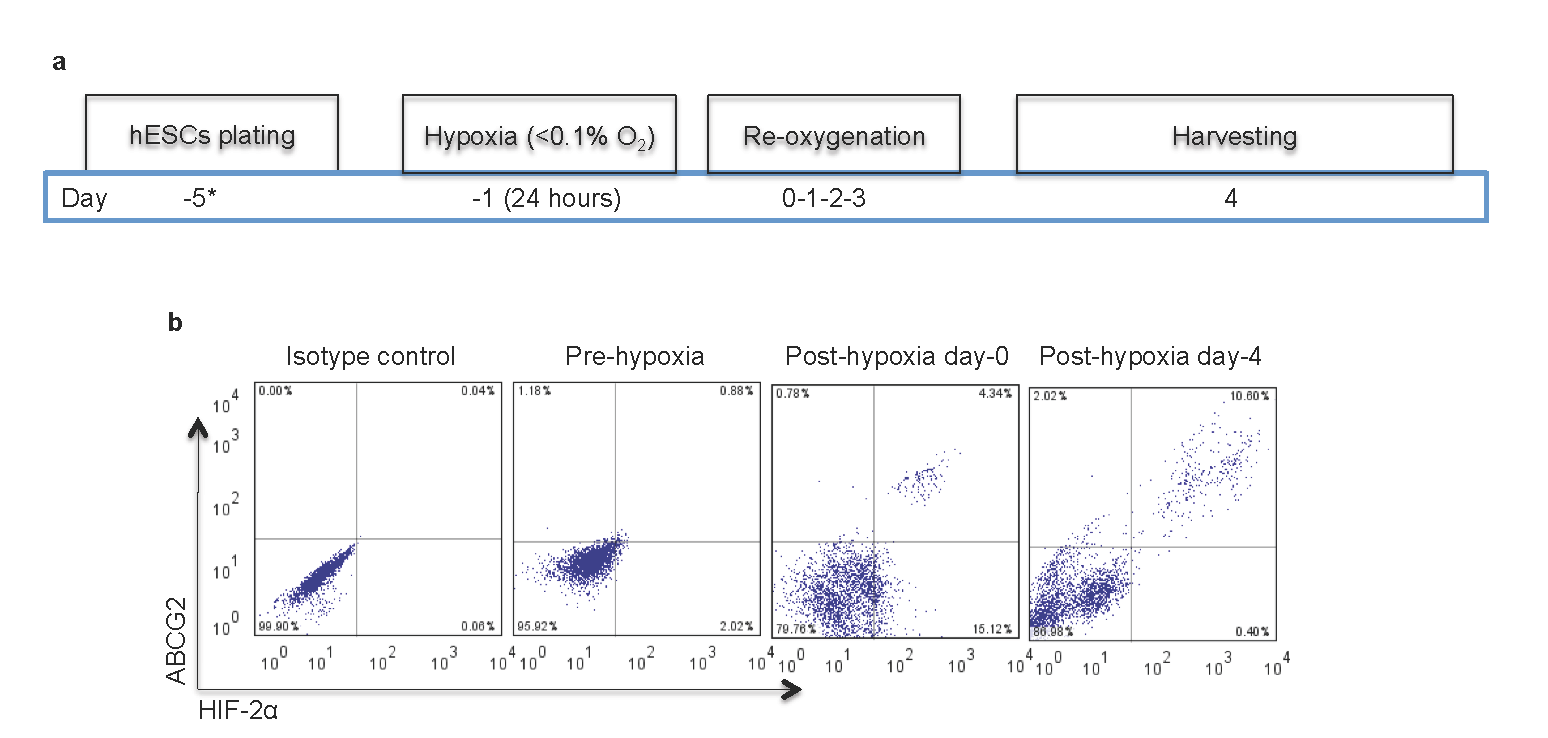

Supplement: Supplementary Figure 1 — (a) A schematic representation of the experimental design of hypoxia/reoxygenation. (b) A representative (n = 4) flow cytometry profile of post-hypoxia BGO1 cells depicting sustained expression of HIF-2α mainly confirmed to the ABCG2+ fraction on day-4 of reoxygenation. The BGO1 hESCs cells were fixed, and stained with both ABCG2 and HIF-2α antibodies, and then counterstained with FITC and PE secondary antibodies respectively. The insert on the top of the far-left flow cytometry profile represents the isotype control. (n = 4; data from 5,000 single-cell events). * hESCs cultures were allowed to grow on average 5-7 days resulting in colonies as depicited Figure 1A (main text). [file stem0030-1685-sd1.tif]

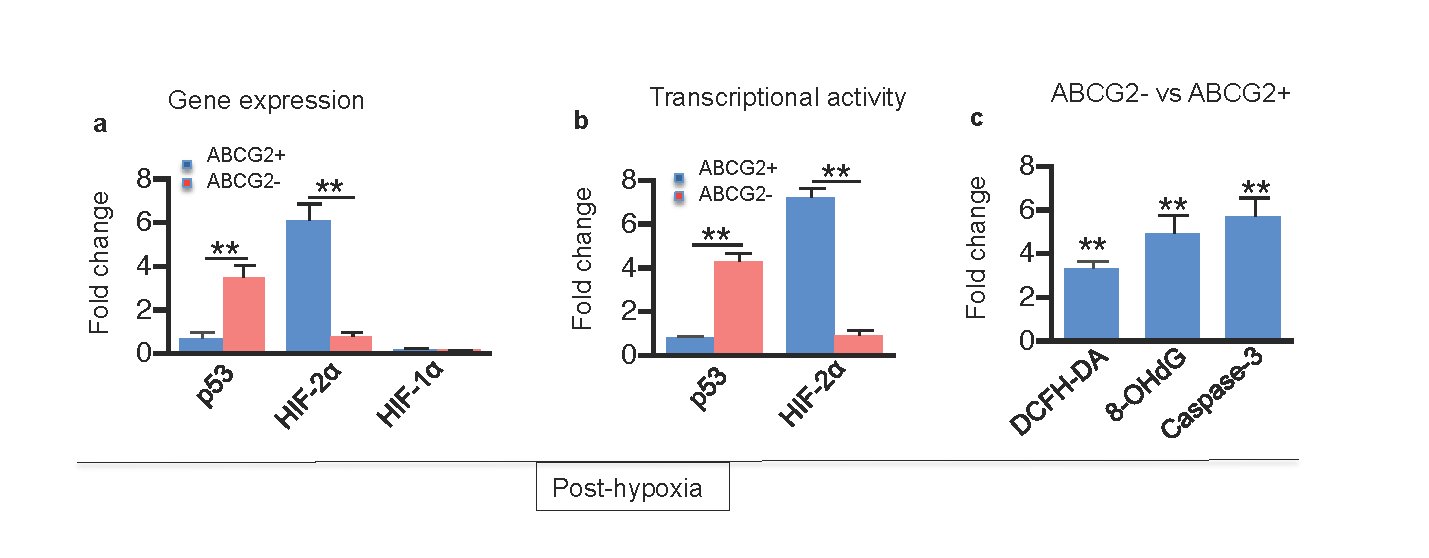

Supplement: Supplementary Figure 2 — (a) Real time PCR (QPCR) data showing the expression of p53, HIF-2α, and HIF-2α expression in the SSEA3+/ABCG2+ and SSEA3+/ABCG2− fractions of BGO1 hESCs between pre-hypoxia and day-4 post hypoxia. QPCR data was first normalized to GAPDH value and then analyzed using – CT method as described22,23 (b) The SSEA3+/ABCG2+ fraction shows high HIF-2α and low p53 activity as measured by the transcriptional activity assay. (c) The ABCG2− versus ABCG2+ fraction of SSEA3+ hESCs showed higher ROS generation, Oxidative DNA damage, and Caspase-3 activity. The BGO1 hESCs cells were exposed to hypoxia, harvested on day-4 re-oxygenation and the values were expressed as the fold change between day-4 in hypoxia. The P < 0.001; n = 3 [file stem0030-1685-sd2.tif]

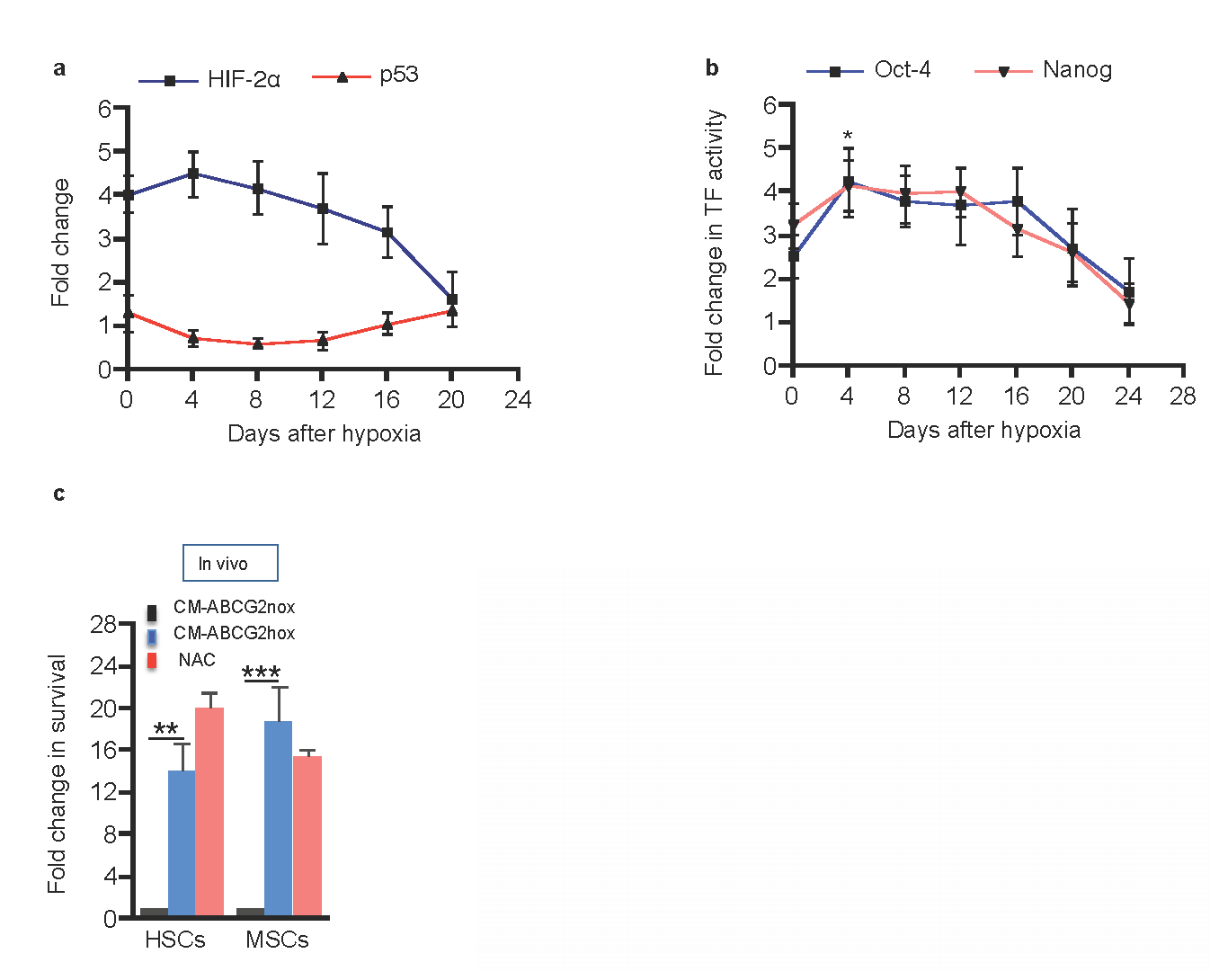

Supplement: Supplementary Figure 3 — H9 derived ABCG2+ hox fraction exhibits highly undifferentiated and cytoprotective state a&b. The fold change in the transcriptional activity of HIF-2α, p53, Oct-4 and Nanog in ABCG2+hox relative to ABCG2+nox cells. c. Fold change in the survival of the mouse HSCs (CD34+ fraction) and MSCs (CD271+ fraction) following treatment of carboplatin-treated mice with conditioned media (CM) derived from the ABCG2+hox versus ABCG2+nox. *p<0.05, **p<0.001, ***<0.0001, n = 3 [file stem0030-1685-sd3.tif]

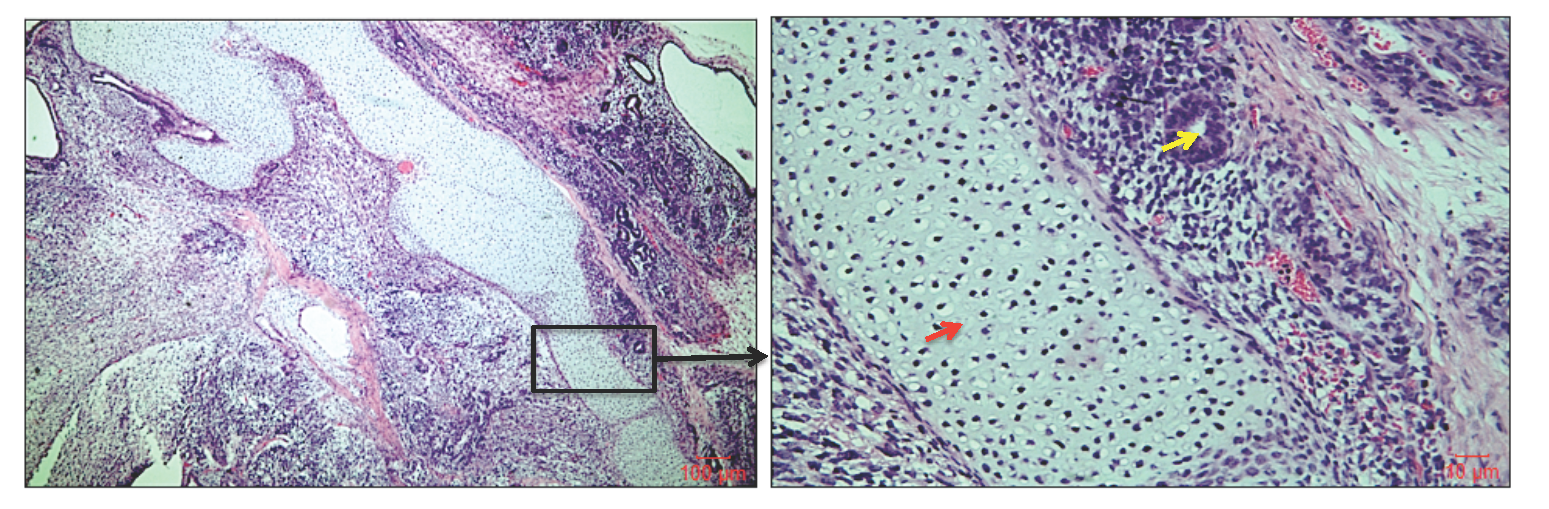

Supplement: Supplementary Figure 4 — In vivo pluripotency of ABCG2+hox cells. The right panel shows an enlarged area of the left panel (Square box) depicting immature cartilage (Red arrow), and gastrointestinal glands (Yellow arrow). ABCG2+hox cells (1x10^4^ cells) were injected to NOD/SCID mice subcutaneously as described23, and a representative 1.5 cm size teratoma was subjected to histological examination by performing H&E staining. [file stem0030-1685-sd4.tif]

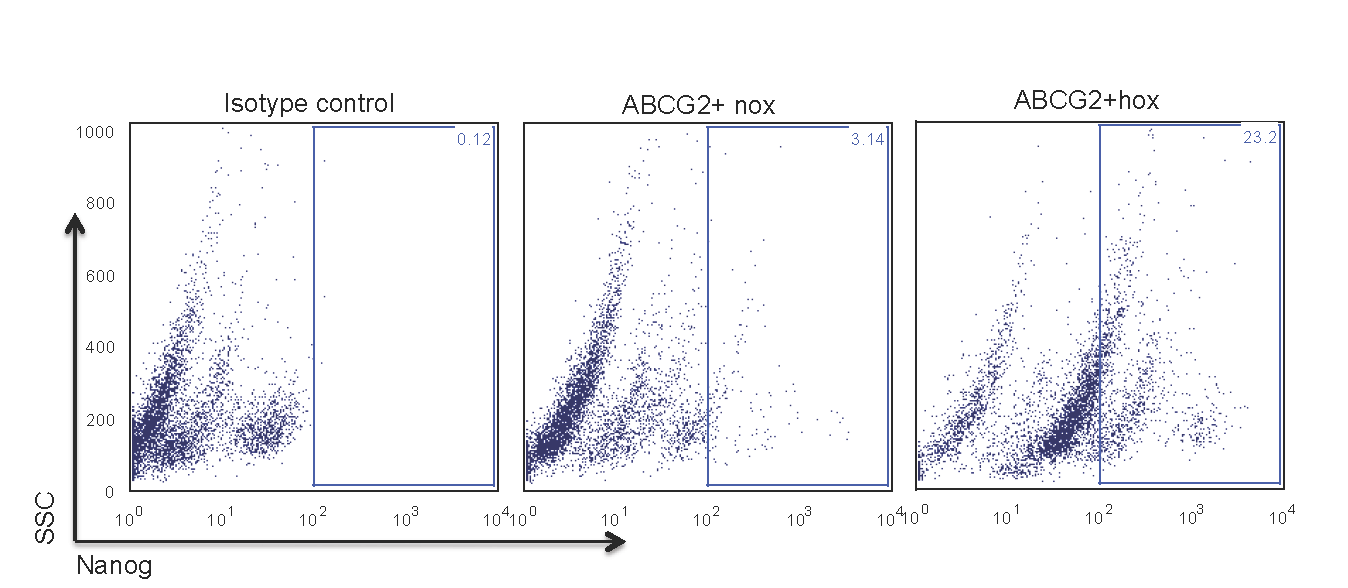

Supplement: Supplementary Figure 5 — Representative flow cytometry images that quantify results presented in Figure 4d, main text. The 10 day old subcutaneous matrigel plugs (n = 5) containing BGO1 hESCs derived SSEA3+/ABCG2+ cells [obtained either before hypoxia (ABCG2+nox) or after day-4 post-hypoxia (ABCG2+hox)] were subjected to enzymatic dissociation 22 and staining with antibody against Nanog28 followed by flow cytometry (5,000 single-cell events were collected). Whereas the ABCG2+nox matrigel plugs contained 3.1 ± 0.5 percentage of Nanog positive cells, the ABCG2+hox matrigel plugs contained 23.5 ± 1.2 percentage of Nanog positive cells (n = 5, *p<0.0001). [file stem0030-1685-sd5.tif]

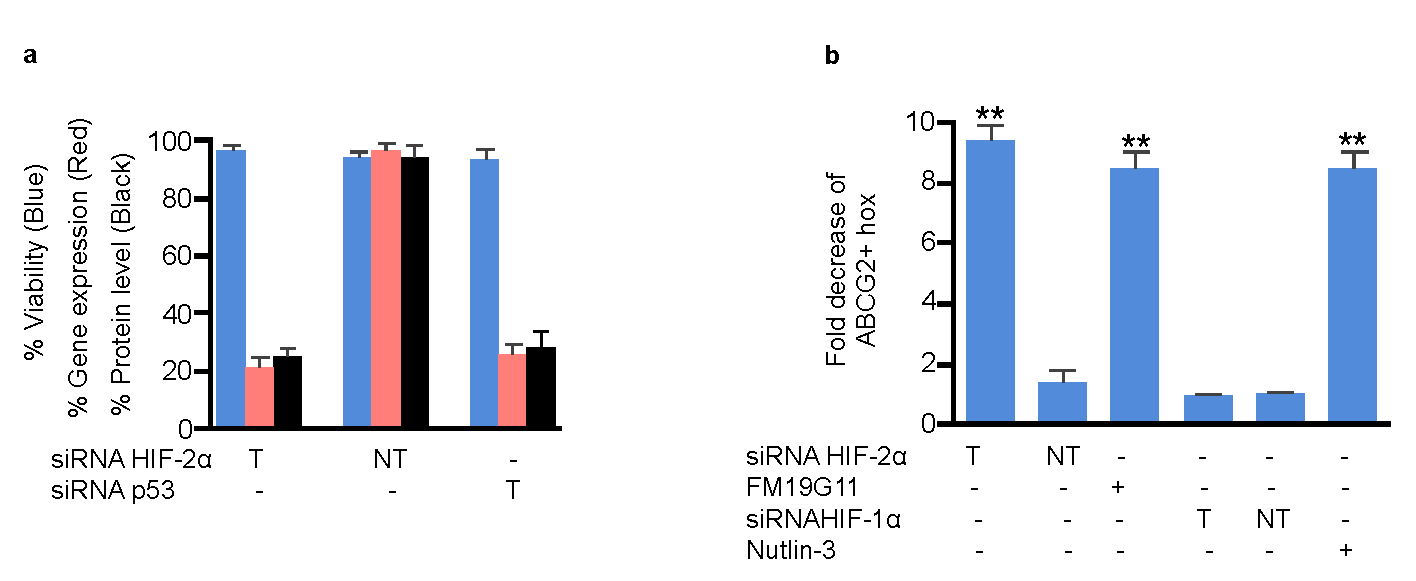

Supplement: Supplementary Figure 6 — (a) QPCR and ELISA data showing the silencing of HIF-2α and p53 by Accell siRNA in ABCG2+hox. The viability tested by Trypan blue showed no significant toxicity following either with targeted (T) or non-targeted (NT) siRNA treatment. The HIF-2α and p53 silencing were assessed by QPCR and ELISA respectively 72-hours after the addition of Accell siRNA 1–M to ABCG2+hox cells (day-4 post hypoxia). The data was compared to the untreated cells. QPCR data was first normalized to GAPDH value and then analyzed using — CT method as described22,23 (b) Fold reduction of post-hypoxia (day 4) ABCG2+ hox cells following siRNA HIF-2α or FM19G11 (0.5–M/ 48 hours) or Nutlin-3 (10–M/48 hours) treatment compared to untreated BGO1 cells. The BGO1 cells treated with above mentioned siRNA or inhibitors were exposed to hypoxia, and the percentage of ABCG2+hox was evaluated by flow cytometry on day-4 post hypoxia. Note that siRNA HIF--α silencing showed no detectable changes in the percentage of ABCG2+hox. n=3. **p<0.001. We achieved HIF-α silencing of >80% protein level by ELISA (not shown). [file stem0030-1685-sd6.tif]

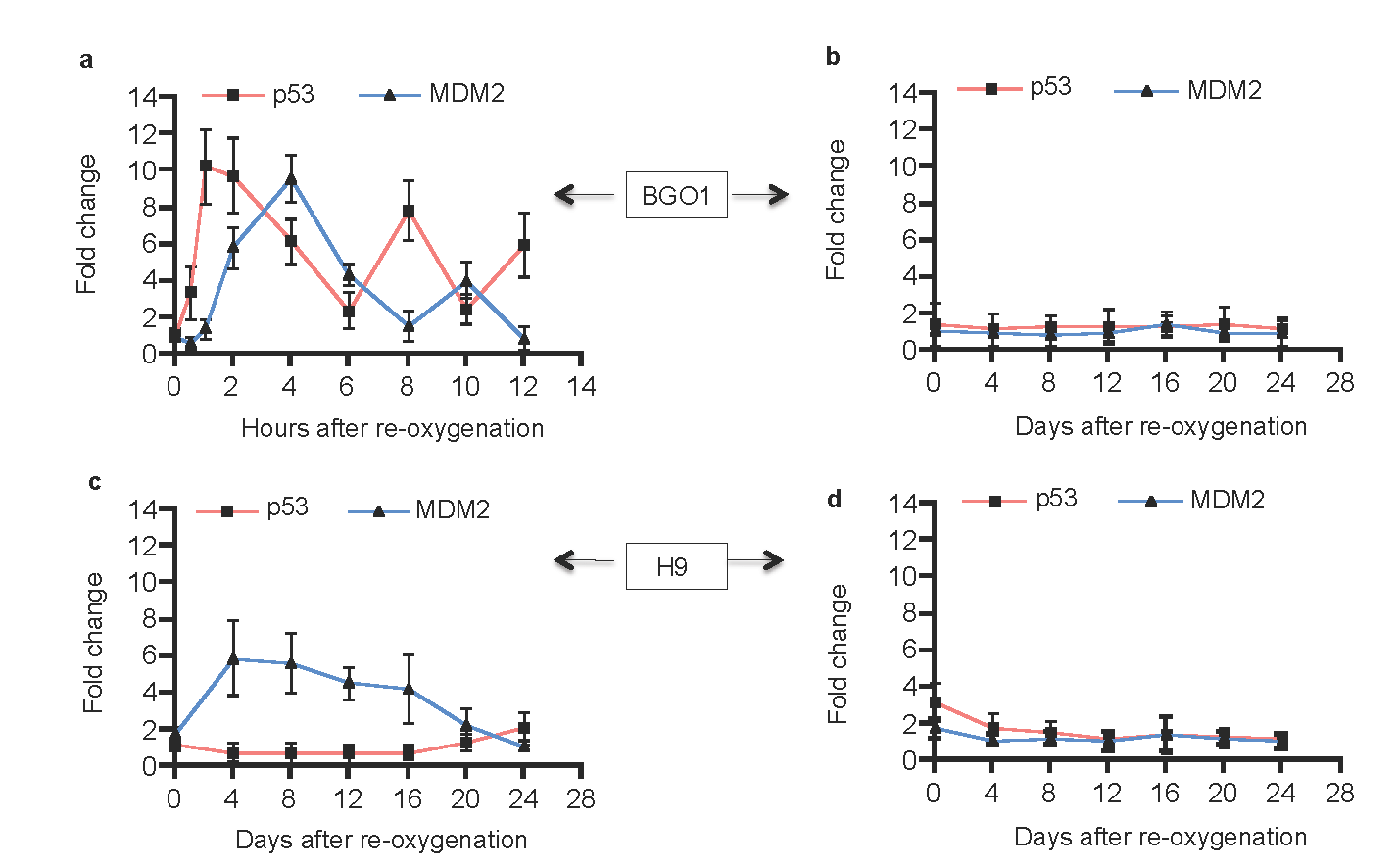

Supplement: Supplementary Figure 7 — (a) The kinetic changes of the levels of p53 and MDM2 proteins (as measured by InCell western) depicted as induction fold in the SSEA3+/ABCG2− fraction immediately and (b) days after hypoxia/reoxygenation. (c) The kinetic changes of the levels of p53 and MDM2 proteins (as measured by ELISA) depicted as induction fold in the hESC line H9 derived SSEA3+/ABCG2+ fraction days after exposure to hypoxia, and (d) represent the state of p53/MDM2 kinetic changes of (c) when the siRNA HIF-2α treated H9 cells were exposed to hypoxia. Data presented represent the mean of four experiments (a&b) and three experiments (c & d). [file stem0030-1685-sd7.tif]

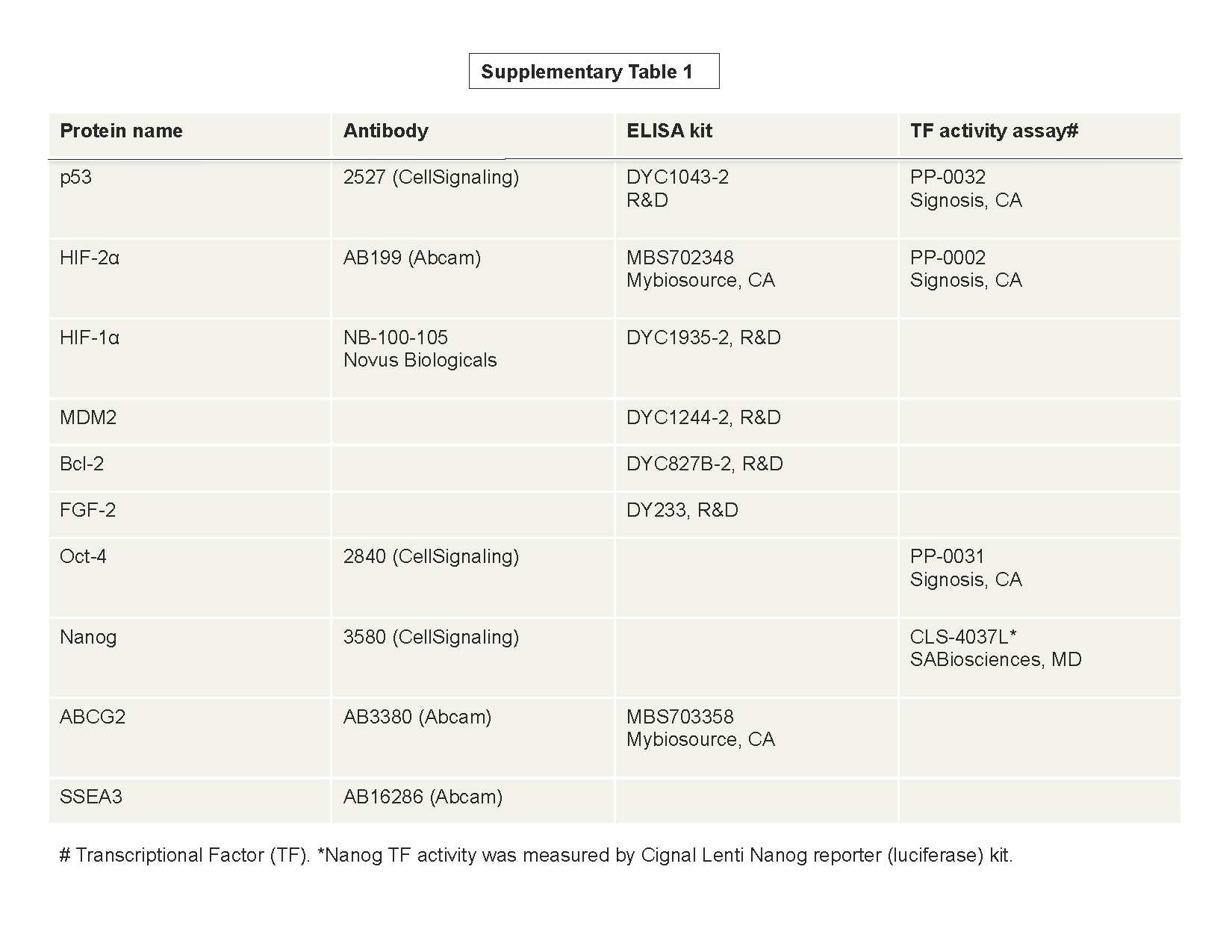

Supplement: Supplementary file 8 — Supplementary Table 1 [file stem0030-1685-sd8.tif]
